# A Transcriptome-Wide Screen for mRNAs Enriched in Fetal Leydig Cells: CRHR1 Agonism Stimulates Rat and Mouse Fetal Testis Steroidogenesis

**DOI:** 10.1371/journal.pone.0047359

**Published:** 2012-10-25

**Authors:** Erin N. McDowell, Anne E. Kisielewski, Jack W. Pike, Heather L. Franco, Humphrey H-C. Yao, Kamin J. Johnson

**Affiliations:** 1 Nemours Biomedical Research, Alfred I. DuPont Hospital for Children, Wilmington, Delaware, United States of America; 2 Reproductive Developmental Biology Group, Laboratory of Reproductive and Developmental Toxicology, National Institute of Environmental Health Sciences (NIEHS/NIH), Research Triangle Park, North Carolina, United States of America; University Hospital of Münster, Germany

## Abstract

Fetal testis steroidogenesis plays an important role in the reproductive development of the male fetus. While regulators of certain aspects of steroidogenesis are known, the initial driver of steroidogenesis in the human and rodent fetal testis is unclear. Through comparative analysis of rodent fetal testis microarray datasets, 54 candidate fetal Leydig cell-specific genes were identified. Fetal mouse testis interstitial expression of a subset of these genes with unknown expression (*Crhr1*, *Gramd1b*, *Itih5*, *Vgll3*, and *Vsnl1*) was verified by whole-mount *in situ* hybridization. Among the candidate fetal Leydig cell-specific factors, three receptors (CRHR1, PRLR, and PROKR2) were tested for a steroidogenic function using *ex vivo* fetal testes treated with receptor agonists (CRH, PRL, and PROK2). While PRL and PROK2 had no effect, CRH, at low (approximately 1 to 10) nM concentration, increased expression of the steroidogenic genes *Cyp11a1, Cyp17a1, Scarb1, and Star* in GD15 mouse and GD17 rat testes, and in conjunction, testosterone production was increased. Exposure of GD15 fetal mouse testis to a specific CRHR1 antagonist blunted the CRH-induced steroidogenic gene expression and testosterone responses. Similar to *ex vivo* rodent fetal testes, ≥10 nM CRH exposure of MA-10 Leydig cells increased steroidogenic pathway mRNA and progesterone levels, showing CRH can enhance steroidogenesis by directly targeting Leydig cells. *Crh* mRNA expression was observed in rodent fetal hypothalamus, and CRH peptide was detected in rodent amniotic fluid. Together, these data provide a resource for discovering factors controlling fetal Leydig cell biology and suggest that CRHR1 activation by CRH stimulates rat and mouse fetal Leydig cell steroidogenesis *in vivo*.

## Introduction

Identifying mechanisms regulating fetal Leydig cell differentiation and function is important for understanding hormone-dependent male reproductive development. Development of the male reproductive system begins with the specification of the testis from the indifferent gonad, through the upregulation of the SRY (sex-determining region Y) gene in Sertoli cell precursors [1], [2], [3]. This upregulation leads to differentiation of Sertoli cells, expression of SOX9 (SRY-box 9) in Sertoli cells, and the subsequent differentiation of Leydig cells through Sertoli-based paracrine signaling [4], [5]. In the mouse, fetal Leydig cells are derived from progenitor cells found in two distinct spatial sites, the coelomic epithelium and the gonad-mesonephros border [6]. NR5A1 (also known as SF1) expression drives Leydig cell differentiation from progenitor cells, while Notch signaling appears to maintain Leydig cell progenitors in an undifferentiated state [7], [8]. Leydig cell differentiation is enhanced by desert hedgehog (DHH) secretion from Sertoli cells [9]. DHH protein binds to the patched 1 receptor on progenitor Leydig cells leading to activation of GLI proteins, with subsequent differentiation of progenitor cells into fetal Leydig cells [9], [10], [11]. Once Leydig cells are differentiated, steroidogenesis is initiated, leading to masculinization of the fetal male reproductive tract and external genitalia.

From approximately gestational weeks (GW) 8 to 10 in the human male fetus, steroidogenesis is controlled by signaling through the LH receptor. Initially, placental-derived chorionic gonadotropin (hCG) binds to the LH/CG receptor (LHCGR) on fetal Leydig cells to enhance steroidogenesis. Once hCG begins to decline, luteinizing hormone (LH) expression from the anterior pituitary begins to increase, driving testosterone production [12]. Although LHCGR activation is required for human fetal testis steroidogenesis from about GW8–10 and onward, human fetal Leydig cell steroidogenesis commences at approximately GW6, and this early period from GW6 to GW8–10 may be independent of LHCGR activation [12], [13]. The early period of fetal testis steroidogenesis from approximately GW8 to GW14, which encompasses both the LHCGR-independent and LHCGR-dependent phases, is known as the masculinization programming window and is imperative for masculinization of the male reproductive tract and external genitalia. In rats and mice, the corresponding window ranges from gestational day (GD) 16 to GD18 and GD14 to GD16, respectively [14].

Unlike humans, the rodent placenta does not produce hCG, and the critical regulator of rodent fetal Leydig cell steroidogenesis during the masculinization programming window is unknown. LHCGR is present in the mouse and rat fetal testis during the masculinization programming window and is capable of stimulating Leydig cell steroidogenesis, but LHCGR is not required for steroidogenesis at this time. LHCGR knockout mice exhibit normal fetal testis steroidogenesis and *in utero* male reproductive development [15]. Not until the production of LH after GD17 in the rat does LHCGR play a necessary role in rat Leydig cell steroidogenesis [12]. Therefore, it remains unknown what drives Leydig cell steroidogenesis at the beginning of the male programming window in humans and what factor(s) is required to activate Leydig cell steroidogenesis during the masculinization programming window in rodents.

To begin closing this knowledge gap, we used a fetal testis comparative genomics approach to identify candidate genes with expression enriched in fetal Leydig cells. From the list, we performed *in situ* hybridization (ISH) to localize a subset of candidate mRNAs in fetal mouse testis and functional tests of candidate receptors in *ex vivo* fetal rodent testes and murine MA-10 Leydig cells to determine potential modulatory activity on steroidogenesis.

## Materials and Methods

### Animals

Timed-pregnant Sprague-Dawley rats and CD-1 mice were purchased from Charles River Laboratories (Raleigh, NC) and housed in the Alfred I. duPont Hospital for Children vivarium. The vivarium is accredited by the Association for Assessment and Accreditation of Laboratory Animal Care International, and all animal care protocols were approved by the Nemours Institutional Animal Care and Use Committee. Rats were housed in polycarbonate cages containing pine shavings, fed Lab Diet Rat Chow 5012 (PMI Nutrition International, Brentwood, MO), and provided with tap water *ad libitum*. Mice were housed in polycarbonate cages with ¼ inch Bed o Cob, fed Lab Diet Mouse Chow 5020 (PMI Nutrition International) and provided with tap water *ad libitum*. The day after mating was defined as GD0.

### Candidate Gene Search

A detailed explanation of this multistep, sequential process is found in the first worksheet of File S1. To discover fetal Leydig cell candidate genes, we analyzed a series of Affymetrix microarray datasets (Table 1). From the GenitoUrinary Molecular Anatomy Project [16], Affymetrix CEL file data were obtained for whole GD11, 12, and 14 mouse testis (Gene Expression Omnibus accession number GSE4818) and GD11, 12, and 13 mouse testis and ovary cell populations isolated by virtue of expressing a fluorescent protein under control of *Mafb*, *Sox9*, or *Pou5f1* promoters (Gene Expression Omnibus accession number GSE27715). Using Bioconductor software packages within the R computing environment, whole testis and isolated cell population data were normalized using GC Robust Multiarray Analysis or Robust Multiarray Analysis, respectively [17], [18], [19], [20], [21], [22]. LIMMA statistical analysis was applied to the normalized expression values [23], [24]. For the dibutyl phthalate (DBP)-exposed rat and mouse testis samples, we used our previously analyzed microarray data [25]. All statistically analyzed microarray data are shown in Files S2, S3, and S4. These array data are all internally consistent when comparing expression patterns of known testis cell-specific genes over developmental time.

**Table 1:** Microarray datasets used to obtain fetal Leydig cell candidate genes.

Species	Sample Type	Fetal Age at Microarray Analysis	Affymetrix Microarray Chip	Group Size	DBP Exposure	Reference
Mouse	*Mafb*+ Testis Cells	GD11, 12, & 13	Gene 1.0 ST	3	Not Applicable	[37]
Mouse	*Sox9*+ Testis Cells	GD13	Gene 1.0 ST	3	Not Applicable	[37]
Mouse	*Pou5f1*+ Testis Cells	GD13	Gene 1.0 ST	3	Not Applicable	[37]
Mouse	*Mafb*+ Ovary Cells	GD13	Gene 1.0 ST	3	Not Applicable	[37]
Mouse	Whole Testis	GD11, 12, & 14	Genome 430 2.0	2–3	Not Applicable	[37]
Mouse	Whole Testis	GD18	Genome 430 2.0	3	Acute	[25]
Mouse	Whole Testis	GD17	Genome 430 2.0	3	Subchronic	[25]
Rat	Whole Testis	GD19	Expression 230 A/B	3–4	Acute	[25]
Rat	Whole Testis	GD19	Genome 230 2.0	3	Subchronic	[25]

The amount of cell contamination in the GD13 *Mafb*-, *Sox9*-, and *Pou5f1*-positive cell isolates was estimated by examining the expression levels of known Leydig, Sertoli, and gonocyte specific genes in each fraction (Figure S1). Little Leydig or Sertoli cell contamination was observed in the *Pou5f1*-positive gonocyte fraction. For the *Mafb*- and *Sox9*-positive cell isolations, the *Mafb*-positive fraction showed modest contamination with Sertoli cells and vice versa, but there was clear enrichment in Leydig and Sertoli cell genes in the appropriate factions.

**Figure 1:**
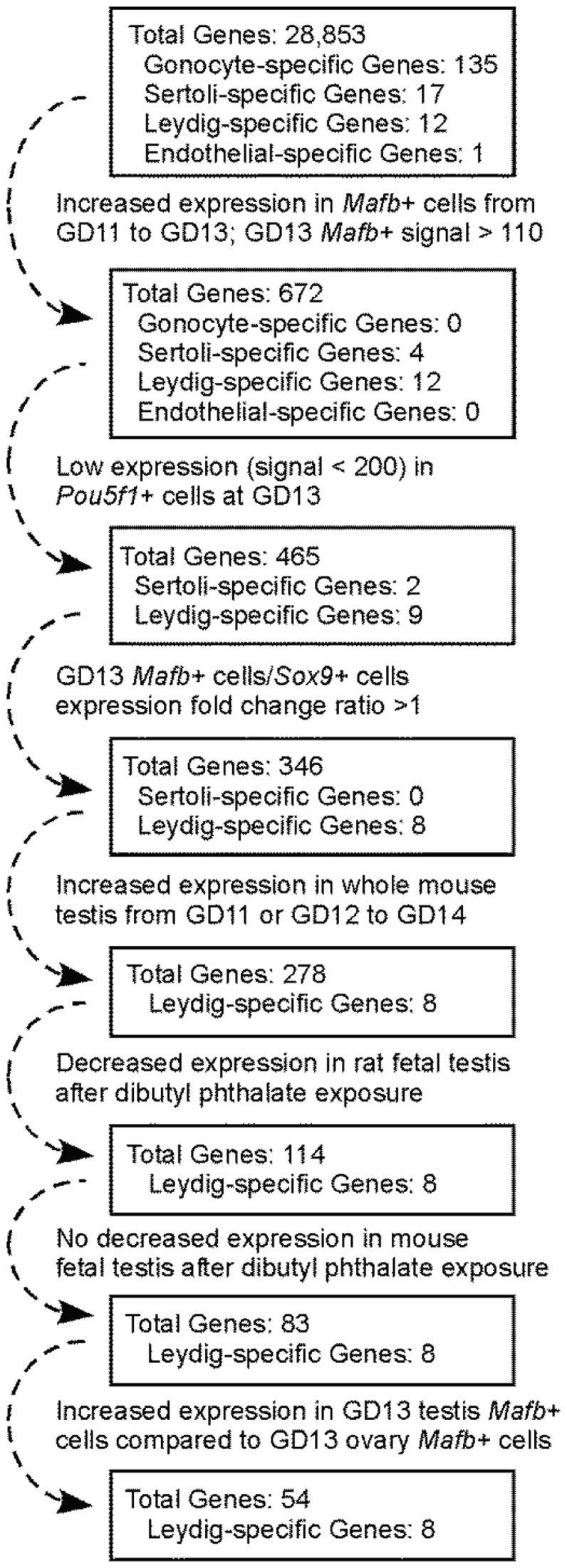
Flowchart showing the steps used to generate the fetal Leydig cell-specific candidate gene list. A detailed explanation of this multistep, sequential process is found in the first worksheet of File S1. In step 1, genes were retained that displayed a signal >110 in GD13 testis *Mafb*-positive cells and an increased expression along with a FDR-corrected p-value <0.25 when comparing GD13 testis *Mafb*-positive cells to GD11 testis *Mafb*-positive cells. In step 2, genes were discarded showing an expression signal >200 in GD13 testis *Pou5f1*-positive cells. In step 3, genes were culled that had higher expression (expression ratio >1) in GD13 testis *Sox9*-positive cells compared to GD13 testis *Mafb*-positive cells. In step 4, genes were retained that showed an increased expression (expression ratio >1) and a FDR-corrected p-value <0.25 when comparing GD11 or GD12 to GD14 whole mouse testis. In step 5, genes were retained showing a decrease in gene expression (expression ratio <1) and a FDR-corrected p-value <0.25 after rat DBP exposure. In step 6, genes were culled that displayed reduced expression (expression ratio <1) and a FDR-corrected p-value <0.25 after mouse DBP exposure. In step 7, genes were retained that showed an increase in expression (expression ratio >1) and a FDR-corrected p-value <0.25 in GD13 testis *Mafb*-positive cells compared to GD13 ovary *Mafb*-positive cells.

To define significant gene expression differences between groups, we set the false discovery rate (FDR)-corrected p-value of a gene to be less than 0.25. This relatively non-stringent cutoff was used because of the relatively small group sizes (n = 3 for most groups) of the microarray data and to yield a list of genes that were not unduly influenced by any one step in this multi-step process (Figure 1). The normalized expression signal cutoffs in steps 1 and 2 were determined by analyzing the expression values of known gonocyte-specific genes in the GD13 *Mafb*-positive cell isolate (signals were generally under 110) and the GD13 *Pou5f1*-positive cell isolate (signals were generally over 200). Not all genes were present on the 3′ Affymetrix chips used to analyze the whole mouse testis and DBP exposure data; genes not present on these chips were carried over to the next step.

### Whole-Mount *In situ* Hybridization

Clones for each candidate gene were generated using primers and templates found in Table S1. After PCR amplification and gel extraction, PCR fragments were cloned into PCR4-TOPO plasmid (Cat# K4575-01, Invitrogen, Grand Island, NY), and clones were sequenced to verify correct insertion and orientation. ISH was performed as previously described [11]. In brief, gonads with attached mesonephros were dissected from GD13 mice, fixed overnight in 4% paraformaldehyde in PBS, and digested in 10 µg/ml proteinase-K solution. Immediately thereafter, samples were fixed in 4% paraformaldehyde-0.1% glutaraldehyde solution for 20 minutes, washed, and incubated in hybridization buffer consisting of 5× SSC (1× SSC is 0.15 M sodium chloride and 0.015 M sodium citrate), pH 5, 50% formamide, 0.1% CHAPS, 0.1% Tween 20, 1 mg/ml yeast tRNA, 50 ng/ml heparin, and 5 mM EDTA (pH 8) for 2 hours at 65°C. Digoxigenin-labeled RNA probe generated from a cloned PCR fragment was added to the hybridization buffer and incubation continued at 60°C for 12 to 16 hours. Samples were washed with prewarmed hybridization buffer and then with room temperature MABTL (5% maleic acid buffer [MAB], 0.1% Tween 20, and 0.05% Levamisol). After incubation for 2 to 4 hours at room temperature in 20% heat-inactivated sheep serum in MABTL blocking (20 mg/ml, blocking powder; Roche Diagnostics Corp., Indianapolis, IN), samples were incubated overnight at 4°C with alkaline phosphatase-conjugated anti-digoxigenin antibody (1∶1000 dilution). After washing in MABTL, samples were incubated in 20 µl/ml alkaline phosphatase substrate (NBT/BCIP; Roche Diagnostics Corp.) in alkaline phosphatase buffer. The color reaction was stopped at an appropriate intensity by washing the samples in PBS, followed by fixation of the samples in 4% paraformaldehyde. ISH of fetal gonads was repeated twice with similar results each time.

### Fetal Testis Ex Vivo Culture

Rat testes and mouse fetal testes were dissected using a stereomicroscope and placed on a filter insert (Cat #PICM01250, Fisher Scientific) in a 24 well×16 mm plate (one testis/well) containing 300 µl of DMEM complete medium alone (vehicle) or with recombinant rat corticotrophin releasing hormone (CRH; Cat# C3042, Sigma-Aldrich), urocortin 1 (UCN1; Cat# E4480, Spring Bioscience, Pleasanton, CA), human chorionic gonadotropin (hCG; Cat# C1063, Sigma-Aldrich), prolactin (PRL; Cat#605345-21-05, LA Biomedical Research, Los Angeles, CA), prokineticin 2 (PROK2; Cat# 100-46, Peprotech, Rocky Hill, NJ), or NBI27914 (CRH antagonist) (Cat# N3911, Sigma-Aldrich). Specific concentrations can be found in the figures or figure legends and the peptide concentrations used to approximate the dissociation constants for their receptors [26], [27], [28], [29], [30], [31], [32], [33]. DMEM complete medium consisted of Dulbecco modified Eagle medium: Nutrient mixture F12 (DMEM:F12) (Cat# 21041025, Invitrogen, Grand Island, NY) supplemented with 1× insulin, transferrin, selenium solution (Cat# I3146, Sigma-Aldrich), 1 mM sodium pyruvate (Cat# S8636, Sigma-Aldrich) and 25 mM HEPES pH7.4 (Cat# H0887, Sigma-Aldrich). Testes were incubated at 37°C in 5% carbon dioxide for 3 or 24 hours. After each time point, media and testes were immediately collected and frozen in microcentrifuge tubes for testosterone and mRNA quantification.

### MA-10 Cell Line Culture

The MA-10 cell line, a murine postnatal Leydig tumor cell line, was a gift from Dr. Mario Ascoli [34]. Cells were grown in DMEM:F12 supplemented with 20 mM HEPES pH7.4, 50 µg/ml gentamicin (Cat# G1264, Sigma-Aldrich), and 15% horse serum (Cat# 26050, Invitrogen). For CRH time course and concentration response experiments, MA-10 cells were plated in 24 well×16 mm plates on Day 0 at a density of 1.25×10^5^ cells/well in a total medium volume of 1 ml. Cells were maintained in a humidified atmosphere with 5% carbon dioxide at 37°C. On Day 1, medium was changed, and cells were cultured until Day 3. On Day 3, cells were washed with 1 ml warm serum-free medium supplemented with 1 mg/ml bovine serum albumin (Cat# A8412, Sigma-Aldrich) (assay medium). Cells were then incubated with 500 µl of assay medium alone or assay medium supplemented with CRH for 1, 3, 6, or 24 hours. After each time point, media and trypsinized cells were collected in microcentrifuge tubes and frozen in −80°C for progesterone and mRNA quantification.

### Testosterone and Progesterone Quantification

Culture medium was sent to the University of Virginia Center for Research in Reproduction Ligand Assay and Analysis Lab for analysis using radioimmunoassays for testosterone (Catalog #TKTT2, Siemens Medical Solutions Diagnostics, Los Angeles, CA; intra-assay coefficient of variation −3.6%, sensitivity −0.1 ng/ml) and progesterone (Cat#TKPG2, Siemens Healthcare Diagnostics, Tarrytown, NY; intra-assay coefficient of variation −5.4%, sensitivity −0.1 ng/ml). Each assay was performed in duplicate.

### Quantitative Reverse Transcription-Polymerase Chain Reaction (qRT-PCR)

Total RNA purification, complementary DNA generation, Taqman-based quantitative PCR, and data analysis using the delta-delta threshold cycle method were performed as described by Barthold et al., 2008 [35]. The amplifications were performed in duplicate, and *Tbp* mRNA levels were used as the endogenous control. The validated Taqman assays (Applied Biosystems Inc., Foster City, CA) used are found in Table S2.

### CRH Quantification In Amniotic Fluid

Amniotic fluid associated with GD15 mouse and GD17 rat fetuses was isolated with a 1 ml syringe, male and female fluid pooled within a litter, and fluid stored at −80°C. Thawed fluid was centrifuged at 20,000×g for 1 min, and CRH within the supernatant was quantified using a fluorescent enzyme immunoassay (Cat# FEK-019-06; Phoenix Pharmaceuticals, Inc., Burlingame, CA). Standards and samples were assayed in duplicate. Data were analyzed using four parameter logistics within Graphpad Prism 5.0 software (Graphpad Software Inc., La Jolla, CA). The sensitivity of the assay was 4.5 pM, and the intra-assay coefficient of variation was 3.3%.

### Statistical Analysis

Statistical significance of all non-microarray data was determined using Graphpad Prism 5.0 software and a one-way ANOVA with Dunnett's Multiple Comparison test for groups of three or more or a two-tailed unpaired t-test for comparison between two groups. Any p-value greater than 0.05 was considered significant.

## Results

### Candidate Fetal Leydig Cell Genes

We based the search for fetal Leydig cell-specific candidate genes upon the following information. In the mouse, fetal Leydig cells are specified and testis steroidogenesis begins at GD12, and testis steroidogenesis increases greatly between GD12 and GD13 [8]. Fetal Sertoli cells and gonocytes specifically express *Sox9* and *Pou5f1*, respectively, while fetal interstitial cells enriched in Leydig cells express *Mafb* [6]. Exposure of fetal rats to DBP profoundly reduces expression of most genes related to differentiated Leydig cell function, including most of the genes known to function in Leydig cell hormone production [25]. However, DBP exposure of fetal mice does not reduce expression of Leydig cell-specific genes [25]. Finally, expression of steroidogenic genes is higher in fetal testis compared to fetal ovary. Thus, the characteristics of a Leydig cell-specific candidate gene would be: 1) higher testis expression at GD13 compared to GD11; 2) expression in *Mafb*+ cells but not *Sox9*+ or *Pou5f1*+ cells; 3) reduced expression in fetal rat testis but not fetal mouse testis following DBP exposure; and 4) increased expression in GD13 fetal testis compared to GD13 fetal ovary.

To discover genes with these characteristics, a comparative microarray analysis of over 28,000 genes was performed (Figure 1). The lists of genes and associated microarray data at each step in the process are shown in File S1. From this analysis, we obtained a list of 54 fetal Leydig cell candidate genes (Table 2). Of the 135 gonocyte-specific and 17 Sertoli cell-specific genes present in the starting gene list, none were present in the final Leydig cell candidate gene list. Known Sertoli- and gonocyte-specific genes were culled midway through the gene selection process. The candidate gene list included 8 of 12 known fetal Leydig cell-specific genes (e.g. *Star*, *Cyp11a1*, and *Lhcgr*), demonstrating a clear enrichment of Leydig-specific genes. Leydig cells possess a high rate of lipid metabolism, and genes functioning in fatty acid or cholesterol synthesis pathways were present in the candidate gene list, including *stearoyl-CoA desaturase 1* (*Scd1*), *fatty acid desaturase 1* (*Fads1*), and *transmembrane 7 superfamily member 2* (*Tm7sf2*).

**Table 2:** List of genes highly enriched in fetal Leydig Cells.

Gene	Fold Change	Gene	Fold Change	Gene	Fold Change	Gene	Fold Change
***Cyp17a1***	43.8	*Thbd*	4	*Robo2*	1.9	*Dlc1*	1.5
***Cyp11a1***	24.8	*Inha*	3.8	*Abcc9*	1.9	*Tm7sf2*	1.5
***Gsta2***	12.5	*Ltbp4*	3.5	*Srpx2*	1.9	*Glipr2*	1.5
*Inhba*	10.9	*Vgll3*	3.5	*Npy*	1.9	*Lrrk2*	1.4
***Hsd3b1***	10.1	*Pi15*	3.3	*Itm2a*	1.8	*Crhr1*	1.4
***Lhcgr***	7	*Prokr2*	3.3	*Gpx3*	1.8	*Cdkn2c*	1.4
***Star***	6.5	*Ppp1r14a*	3.1	*Sec24d*	1.8	*Gucy1b3*	1.4
*Vsnl1*	5.9	*5031410I06Rik*	2.7	*Ng23*	1.8	*Slc29a1*	1.4
*Speer4d*	5.6	***Aebp1***	2.7	*Htra3*	1.6	*1200009O22Rik*	1.3
*Gramd1b*	4.2	*Cd36*	2.3	*4930474M22Rik*	1.6	***Insl3***	1.3
*Prlr*	4.2	*Alcam*	2.2	*PtrfB*	1.6	*Fads1*	1.3
*AI427809*	4.2	*Gria4*	2.1	*B3galt1*	1.5	*Arx*	1.3
*Itih5*	4.1	*Nuak1*	2.1	*Scd1*	1.5	*Admts7*	1.3
*Itgb8*	4.1	*Fbn1*	2.1				

Genes in bold are known fetal Leydig cell-specific genes. Fold change values are the increase in mRNA levels from GD11 to GD13 in *Mafb+* cells.

**Figure 2:**
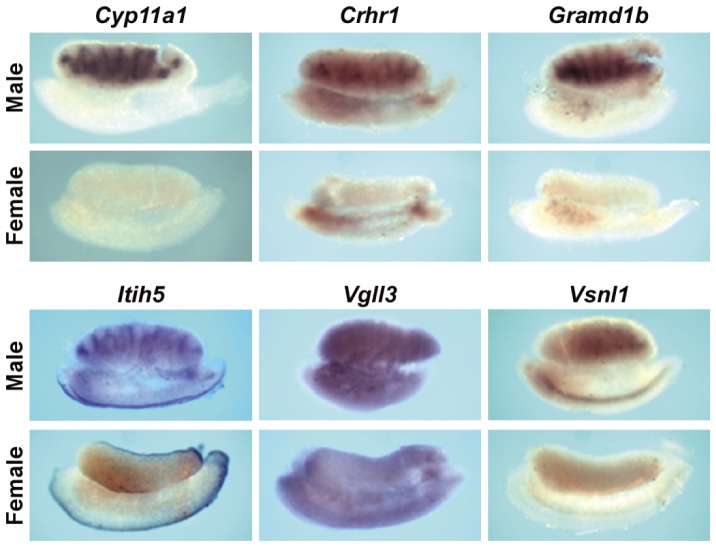
Fetal mouse gonad whole-mount ISH of select candidate Leydig cell-specific genes. Shown are ISH patterns of GD13 mouse gonads with attached mesonephros below the gonad. The distribution of fetal testis Leydig cells is shown in the male *Cyp11a1* image.

To gauge the selectivity of the candidate gene list for fetal testis expression in the interstitial compartment containing Leydig cells, we used fetal gonad *in situ* hybridization. We localized a subset of candidate genes with unknown expression patterns in fetal testis: *corticotropin releasing hormone receptor 1* (*Crhr1*), *GRAM domain containing 1B* (*Gramd1b*), *inter-alpha (globulin) inhibitor H5* (*Itih5*), *vestigial like 3* (*Vgll3*), and *visinin-like 1* (*Vsnl1*). These genes were chosen because of their involvement in a range of regulatory pathways [cAMP signaling (*Crhr1*); extracellular peptidase inhibition (*Itih5*); transcription (*Vgll3*); and calcium signaling (*Vsnl1*)], their unknown function in fetal testis, their range of expression levels in GD13 *Mafb*+ cells (from a low of 178 for *Crhr1* to a high of 942 for *Gramd1b*), and/or their range of fold change induction in *Mafb*+ cells from GD11 to GD13 (Table 2). The characteristics of these genes in various microarray datasets are shown in Figures S2 and S3. In GD13 mouse gonads, all genes displayed higher ISH signals in testis compared to ovary (Figure 2). Within the testis, *Crhr1*, *Gramd1b*, *Itih5*, and *Vsnl1* ISH signals were distributed similarly to the known fetal Leydig cell-specific steroidogenic gene *Cyp11a1*, supporting the expression of these genes in fetal Leydig cells. *Vgll3* ISH signal was observed throughout the testis without an apparent specific signal within the interstitium.

### CRHR1 Activation Increases Rat and Mouse Fetal Testis Steroidogenesis During the Masculinization Programming Window

**Figure 3:**
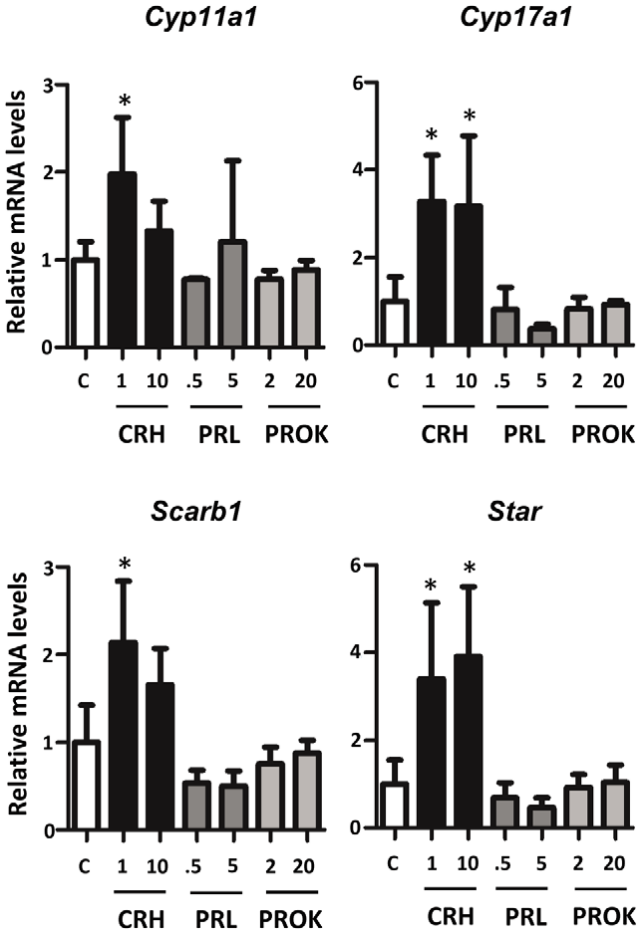
GD17 rat testis steroidogenic gene expression after treatment with CRH, PRL, or PROK2. Testes were exposed for 24 hours *in vitro* to varying concentrations of CRH, PRL, or PROK2. Taqman-based qRT-PCR was used for determination of mRNA levels. Three to four samples per group. Mean ±SD are shown for all data. C: control. Asterisk indicates significance for p-value of <0.05 when compared with controls.

The list of fetal Leydig cell candidate genes contained receptors with the potential to modulate fetal Leydig cell steroidogenesis. To test this possibility, *ex vivo* GD17 rat testes were exposed to agonists of three receptors, corticotropin releasing hormone receptor 1 (CRHR1), prolactin receptor (PRLR), and prokineticin receptor 2 (PROKR2) for 3 and 24 hours. No agonist had a significant steroidogenic gene expression effect after 3 hours of exposure (data not shown). In control testes under basal conditions, steroidogenic gene expression was reduced between 3 and 24 hours of *ex vivo* culturing (data not shown). CRH, a CRHR1 agonist, had a significant effect on steroidogenic genes, *Cyp11a1*, *Cyp17a1*, *Scarb1*, and *Star* through increased gene expression (Figure 3). PRL and PROK2 had no significant effect on steroidogenic gene expression.

**Figure 4:**
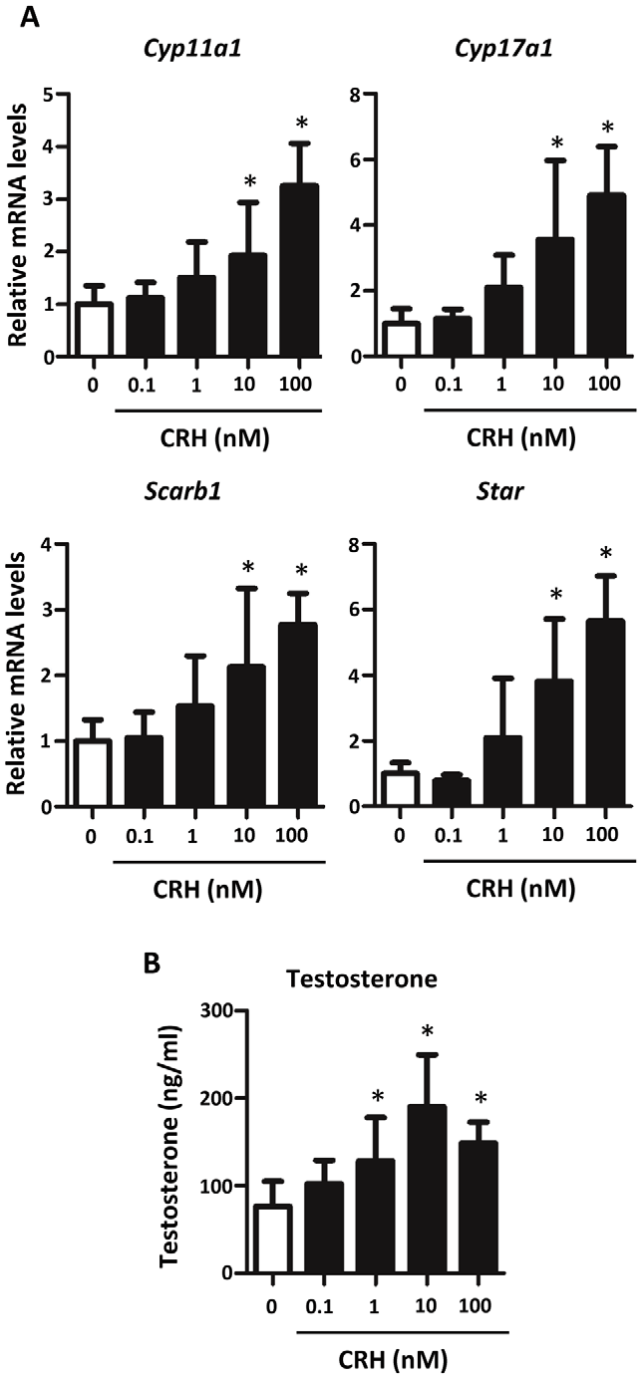
Effects of CRH on steroidogenesis in GD17 rat testis. A) Treatment with CRH increased steroidogenesis in GD17 rat testis. Seven to nineteen testes per group were exposed for 24 hours *ex vivo.* mRNA levels were determined using Taqman-based qRT-PCR. Mean ±SD are shown for all data. Asterisk indicates significance for p-value of <0.05 when compared with controls. B) Testosterone secretion increased in GD17 rat testis treated with CRH. Media were collected from eight to twenty samples per group for testosterone radioimmunoassay analysis. Mean ±SD is shown for all data. An asterisk indicates significance of a p-value <0.05.

**Figure 5:**
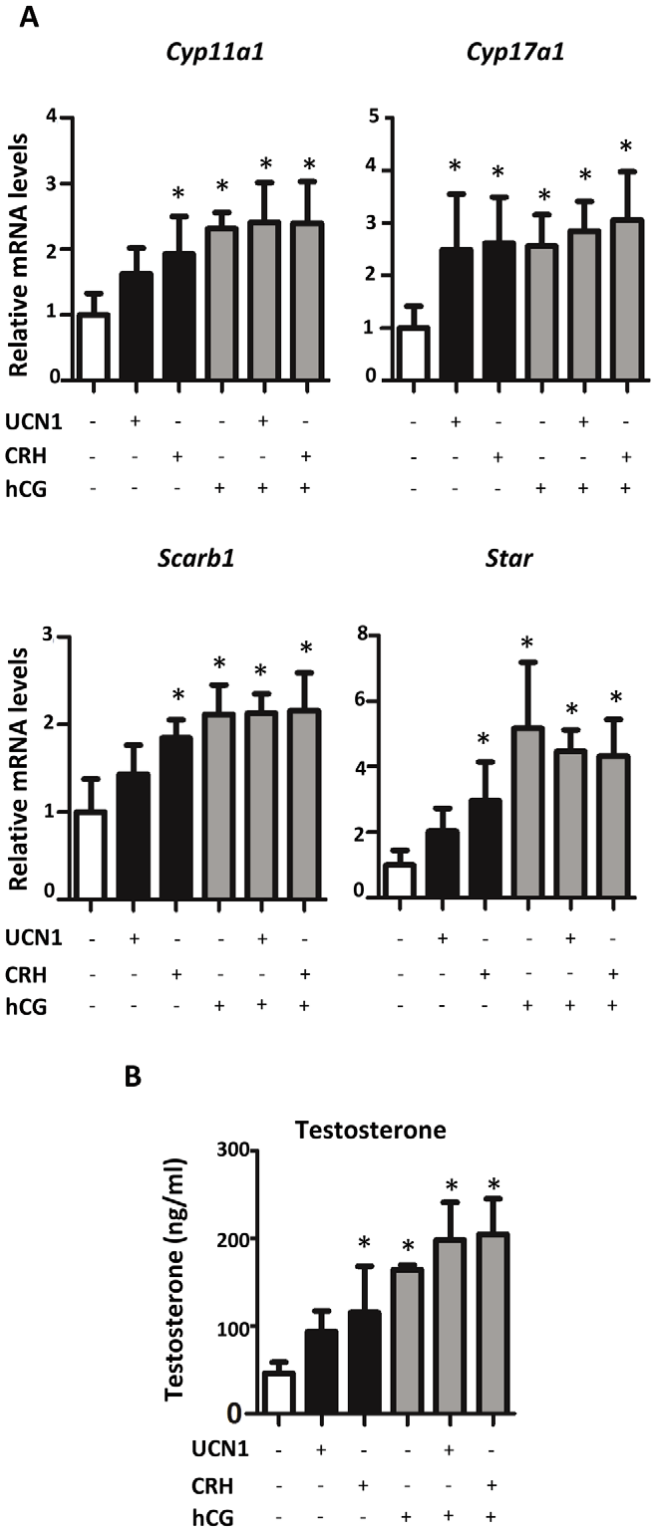
Effects of CRH and UCN1 on steroidogenic gene expression in GD19 rat testis. A) Steroidogenic gene expression in GD19 rat testis after exposure to 1 nM CRH, 1 nM UCN1, and/or 0.1 IU/ml hCG. Testes were exposed for 24 hours, and mRNA levels were determined using Taqman-based qRT-PCR. Mean ±SD are shown for all data. Four to five samples per group. Asterisk indicates significance for p-value of <0.05 when compared with controls. B) Testosterone levels in GD19 rat testis after exposure *in vitro* to 1 nM CRH, 1 nM UCN1, and/or 0.1 IU/ml hCG. Testes were exposed for 24 hours and media collected from four to five samples/group for testosterone measurement. Mean ±SD shown for all data. Asterisk indicates significance for p-value of <0.05 when compared with vehicle control.

Seeing an effect with CRHR1 activation on steroidogenic gene expression, the concentration response of CRH on fetal mouse and rat testis steroidogenic gene expression was examined. GD17 rat testes within the masculinization programming window were exposed *ex vivo* to four differing concentrations of CRH (0.1, 1, 10, and 100 nM) for 24 hours. Steroidogenic pathway gene (*Cyp11a1*, *Cyp17a1*, *Scarb1*, and *Star*) mRNA levels increased in a concentration-dependent manner and were elevated significantly at 10 and 100 nM CRH (Figure 4A). Although not statistically significant, there appeared to be a trend toward increased gene expression at 1 nM CRH. In conjunction with the elevation in steroidogenic gene expression, testosterone levels also increased at CRH concentrations of 1 nM or higher (Figure 4B). *Ex vivo* cultured GD19 rat testes also showed enhanced steroidogenic pathway gene expression when exposed to 1 nM CRH and 1 nM UCN1 (another agonist for CRHR1) for 24 hours (Figure 5A) [33], [36]. When treated with hCG alone, mRNA levels of steroidogenic pathway genes increased. However, no additional increase in *Cyp11a1*, *Cyp17a1*, *Scarb1*, or *Star* mRNA levels was seen when testes were treated with a combination of CRH and hCG (Figure 5A). In addition to the enhanced steroidogenic gene expression, testosterone levels were increased for GD19 testes exposed to 1 nM CRH, hCG, and a combination of hCG and CRH, along with hCG and UCN1 (Figure 5B). However, there was no additional statistically significant increase in testosterone levels when comparing hCG alone to hCG in combination with CRH or UCN1.

**Figure 6:**
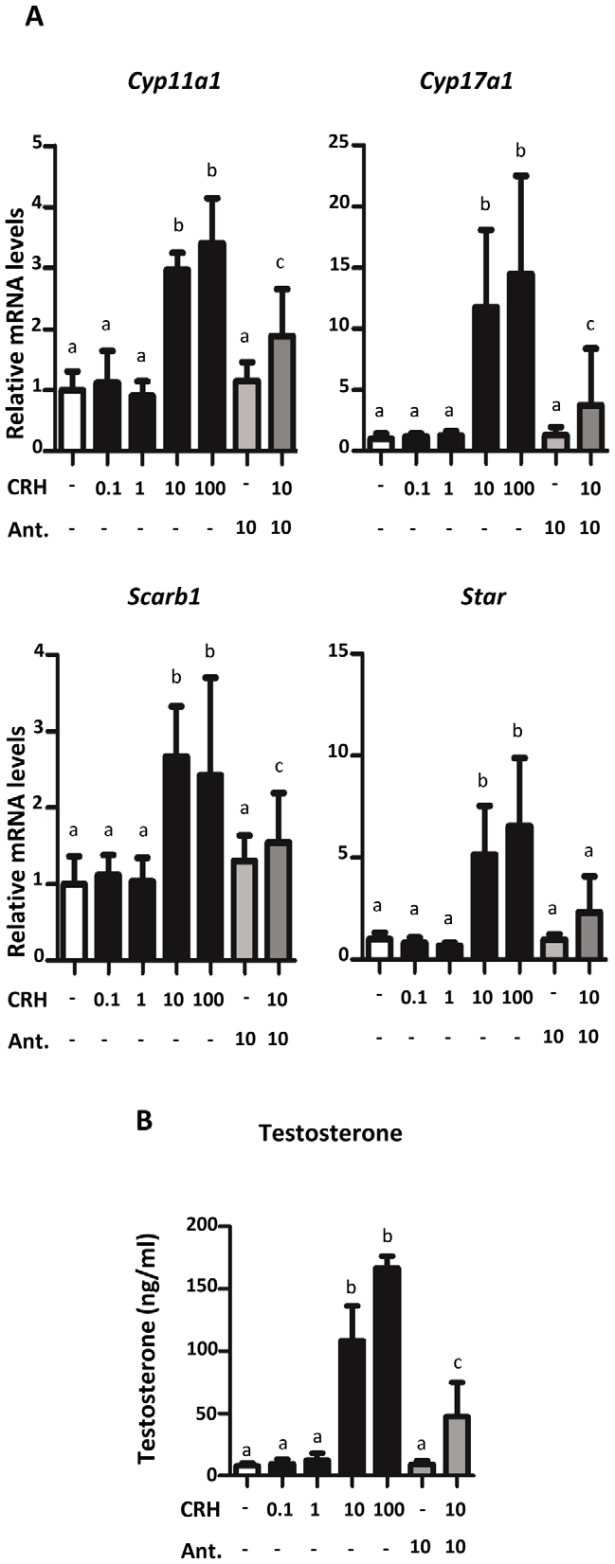
Effects of CRH on steroidogenic gene expression in GD15 mouse testis. A) Steroidogenic mRNA expression increased after treatment with CRH and/or antagonist in GD15 mouse testis. Eight to twenty-one samples/group were exposed for 24 hours *in vitro* to varying concentrations of CRH and/or 10 µM of antagonist. mRNA levels were determined by Taqman-based qRT-PCR. Mean ±SD shown for all data. a indicates no significant change between treated samples and vehicle samples. b indicates a significant increase (p-value <0.05) between treated and vehicle samples. c indicates a significant decrease (p-value <0.05) between samples exposed only to 10 nM CRH and samples exposed to 10 nM CRH and 10 µM CRH antagonist. (Ant. = antagonist) B) CRH treatment increased testosterone secretion from GD15 mouse testis. Testes were exposed to varying concentrations of CRH and/or 10 µM of antagonist. Media were collected from seven to fifteen samples/group for testosterone radioimmunoassay analysis. Mean ±SD shown for all data. a, b, and c are described above.

To determine if CRH stimulates steroidogenesis in fetal mouse testes within the masculinization programming window, GD15 mouse testes were treated with varying concentrations of CRH for 24 hours (Figure 6A). At concentrations of 10 or 100 nM, CRH significantly increased mRNA levels of steroidogenic pathway genes (*Cyp11a1*, *Cyp17a1*, *Scarb1*, and *Star*). When exposed to 10 nM CRH and a specific CRHR1 antagonist (10 µM), the mRNA levels significantly decreased when compared to testes exposed to 10 nM CRH alone. Similar to steroidogenic pathway gene expression, GD15 mouse testis testosterone production increased after incubation with 10 nM or 100 nM CRH, and this CRH-induced increase was significantly decreased by CRHR1 antagonism (Figure 6B). Unlike steroidogenic genes, mRNA levels of the Leydig cell specific gene *Insl3* were unaltered by CRHR1 agonists in GD17 rat and GD15 mouse testes (data not shown).

### CRH Increases Steroidogenesis in MA-10 Cells

**Figure 7:**
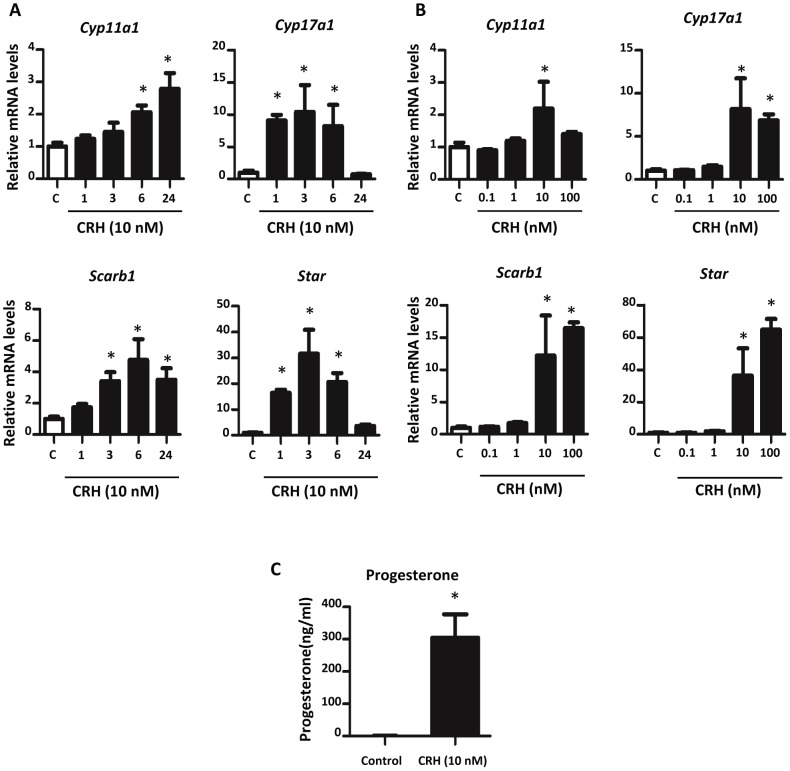
Effect of CRH on MA-10 cell steroidogenesis. A) CRH exposure of MA-10 cells increased steroidogenic gene expression at different time points. MA-10 cells were exposed to 10 nM CRH for 1, 3, 6 or 24 hours. Three to six replicates were analyzed at each time point. Taqman-based qRT-PCR was used to determine mRNA levels. Mean ±SD are shown for all data. Asterisk indicates significance for p-value of <0.05 when compared with controls. B) CRH concentration-response of MA-10 cell steroidogenic mRNA expression. MA-10 cells, five replicates/group, were exposed to varying concentrations of CRH for 6 hours. Taqman-based qRT-PCR was used to determine mRNA levels. Mean ±SD shown for all data. Asterisk indicates significance for p-value of <0.05 when compared with controls. C) CRH increased MA-10 cell progesterone secretion. Eight replicates/group were treated for 6 hours, and progesterone levels in media were quantified by radioimmunoassay. Mean ±SD shown for all data. C: Control. Asterisk indicates significance of a p-value <0.05.

To determine if CRH could directly stimulate Leydig cell steroidogenesis, MA-10 Leydig cells were subjected to 10 nM CRH treatment for 1, 3, 6, or 24 hours. These expression data showed that CRH increased mRNA levels of steroidogenic pathway genes with differing kinetics (Figure 7A). *Cyp17a1* and *Star* mRNA levels were significantly elevated after 1 hr of CRH exposure, but by 24 hours, *Cyp17a1* and *Star* gene expression had waned to control levels. Compared to *Cyp17a1* and *Star*, *Cyp11a1* and *Scarb1* mRNA levels increased significantly at later time points but remained significantly increased after 24 hours. All four genes were significantly increased at the 6 hr time point, and this time point was chosen to analyze the concentration response of CRH exposure. MA-10 cells were exposed to varying concentrations of CRH ranging from 0.1 nM to 100 nM over a 6 hour period (Figure 7B). Only the 10 nM and 100 nM concentrations produced significant elevations in steroidogenic gene expression; 0.1 and 1 nM CRH did not show an effect when compared to controls. At 10 nM CRH, a significant increase in MA-10 cell progesterone production was observed (Figure 7C).

### Expression of CRH and UCN1 in Male Fetal Tissues and Amniotic Fluid

**Figure 8:**
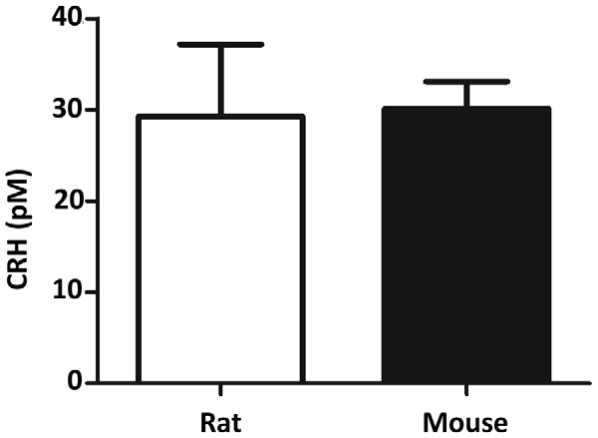
CRH protein levels are detectable in amniotic fluid from GD17 rat and GD15 mouse testis. CRH protein levels in amniotic fluid were measured using a fluorescent enzyme immunoassay. Mean ±SD are shown for all data.

To determine if CRHR1 agonists were expressed in the rodent fetus during the masculinization programming window, potential tissue sources of CRH or UCN1 production in the rodent male fetus were examined. Multiple tissues from GD15 to GD19 rat fetuses were screened for *Crh* and *Ucn1* mRNA expression using qRTPCR (Table 3). Only hypothalamus and whole brain samples expressed detectable levels of *Crh*, and this expression was seen at GD17 and GD19. No fetal rat testis *Crh* or *Ucn1* mRNA expression was observed at any gestational age, and no detectable level of *Ucn1* mRNA expression was found in any fetal rat tissue examined. Demonstrating that CRH peptide was produced in the fetus during the masculinization program window, CRH peptide was observed in amniotic fluid from both GD17 male rats and GD15 male mice (Figure 8).

**Table 3:** Cycle threshold (Ct) values of *Crh* and *Ucn1* mRNA in rat fetal tissues.

Organ	*Crh* Average Ct (SD)	*Ucn1* Average Ct (SD)	Gestational Day
Adrenal gland	ND	ND	19
Bladder	ND	ND	19
Brain	30.1 (0.01)	ND	19
Epididymis	ND	ND	19
Gubernaculum	ND	ND	19
Heart	ND	ND	19
Hypothalamus	32.1 (0.96)	ND	17
Intestine	ND	ND	19
Kidney	ND	ND	19
Liver	ND	ND	19
Lung	ND	ND	19
Pancreas	ND	ND	19
Placenta	ND	ND	15
Placenta	ND	ND	17
Placenta	ND	ND	19
Spleen	ND	ND	19
Stomach	ND	ND	19
Testis	ND	ND	15
Testis	ND	ND	17
Testis	ND	ND	19

ND = Not detectable; Ct value over 34. Applied Biosystems does not recommend using any data with a Ct value over 34 due to poor accuracy.

## Discussion

Using a comparative genomics approach, a list of fetal Leydig cell specific candidate genes was identified. As with any candidate gene search, there are likely false-positives and false-negatives in the gene list. From a starting analysis of over 28,000 genes containing 12 known Leydig cell-specific genes, the final list of 54 candidate Leydig cell-specific genes contained 8 of the known Leydig cell-specific genes. Thus, there is a striking enrichment of Leydig cell genes in the candidate gene list. When the expression of five Leydig cell-specific candidate genes with unknown gonadal expression patterns were localized via ISH in GD13 mouse gonads, all showed higher expression in testis compared to ovary. With the exception of *Vgll3*, the expression pattern of the localized genes was similar to the known Leydig cell-specific gene *Cyp11a1*. Using the same fetal mouse testis cell isolates as used here but not the microarray data from DBP exposed fetal testes or whole fetal testes, Jameson et al., compiled a set of Leydig cell-enriched candidate genes [37]; about 25% of the 54 genes in our candidate gene lists are present within the Jameson gene list. While this concordance is significant, overlap between the two lists was reduced by different statistical criteria and the use of additional filters in our protocol, including whole testis and DBP exposure data. *In utero* DBP exposure reduces the expression of Leydig cell genes involved in INSL3 production and lipid metabolism (cholesterol, fatty acid and steroid biosynthesis; [25]) but does not reduce all Leydig cell-specific genes (such as *Stc1*; [38]). In this light, the candidate list of 54 genes may be enriched in genes involved in Leydig cell lipid metabolism. However, not all 54 candidate genes were screened for expression in Leydig cells. File S1 shows the genes identified at each step in the selection process. These genes are potential factors involved in fetal Leydig cell differentiation or hormone production, and mutation of some may be linked potentially to disorders of human masculinization.

Two particularly interesting candidate genes are *Vsnl1* and *Gramd1b*. VSNL1 is a myristoylated calcium binding protein that regulates intracellular signaling [39], while GRAMD1B is a predicted transmembrane protein with unknown function. By ISH in fetal testis, both appear to be expressed specifically in Leydig cells. Our localization data for *Gramd1b* is corroborated by ISH data from genpaint.org (set identification number ES3003); at GD14 in the mouse, *Gramd1b* mRNA is expressed only in steroidogenic cells of the adrenal gland and testis. In steroidogenic adrenal cells, *Vsnl1* and *Gramd1b* mRNA levels are increased by NR5A1 overexpression [40]. Because NR5A1 activity controls fetal Leydig cell steroidogenesis [41], [42], *Vsnl1* and *Gramd1b* may be downstream effectors of NR5A1 activity in fetal Leydig cells.

For functional studies of fetal Leydig cell-specific candidate genes, we focused on genes encoding receptors that may modify Leydig cell steroidogenesis. Of the three candidate genes we studied, only CRHR1 stimulated fetal testis steroidogenesis in rats and mice. Localized *Crhr1* mRNA expression in the interstitial compartment of GD13 mouse gonads with a pattern similar to the Leydig cell-specific gene *Cyp11a1* suggests CRHR1 is produced specifically in fetal Leydig cells. These *Crhr1* ISH data are corroborated by our extensive comparative microarray analysis. When GD17 rat testes were exposed to varying concentrations of the CRHR1 agonist CRH for 24 hours *ex vivo*, 10 nM and 100 nM concentrations increased mRNA levels of the steroidogenic genes, *Cyp11a1*, *Cyp17a1*, *Scarb1*, and *Star*. While a trend of increased steroidogenic gene expression was seen with 1 nM CRH, this increase was not significant. However, an upregulation of testosterone production was observed at 1 nM and higher CRH. These concentration response data approximate the low nM dissociation constant of CRH for CRHR1 in non-testis cell types [43], [44], [45]. Similar enhancing effects on steroidogenesis by CRH were seen in GD15 mouse testis *ex vivo* cultures. Expression levels of steroidogenic genes increased in testes exposed to 10 and 100 nM CRH. To determine if the increase in steroidogenic mRNA levels was due to CRHR1 receptor activation, GD15 mouse testis was treated with CRH and an antagonist specific for CRHR1, NBI 27914, for 24 hours *ex vivo*. Exposure of testis to 10 nM CRH and 10 µM antagonist decreased steroidogenic gene expression when compared to 10 nM CRH alone, indicating CRH stimulates fetal Leydig cell steroidogenesis through CRHR1 activation. UCN1 also activates CRHR1 [46], and the ability of UCN1 to elevate fetal rat Leydig cell steroidogenic gene expression provides additional evidence supporting a stimulatory role of CRHR1 in fetal Leydig cell steroidogenesis (Figure 5A). CRH is an agonist for both CRHR1 and CRHR2, but it binds with higher affinity to CRHR1 [46]. *Crhr2* mRNA levels were below the level of detection in mouse and rat fetal testes microarrays (data not shown), suggesting CRHR2 is not expressed in fetal rodent testes. From the totality of these data, we conclude that CRH and UCN1 stimulate fetal Leydig cell steroidogenesis through activation of CRHR1.

To date, rat and mouse adult Leydig cells and dispersed Leydig cells from GD20 rats have been used in previous CRH studies examining steroidogenesis. Consistent with our data using fetal rat and mouse testes *ex vivo*, CRH stimulated steroidogenesis in MA-10 mouse Leydig cells and primary adult mouse Leydig cells. In postnatal mouse Leydig cells, CRH stimulates Leydig cell steroidogenesis via a mechanism similar to hCG [47], [48], [49]. In contrast, CRH does not enhance steroidogenesis in primary adult rat Leydig cells [47]. Instead, CRHR1 activation may inhibit hCG-stimulated steroidogenesis in primary adult rat Leydig cells [31], [50]; however, Huang et al., [47] did not observe this inhibitory effect in adult rat Leydig cells. One study examined the steroidogenic effect of CRH on rat Leydig cells isolated from GD20 animals (just prior to parturition). After isolation, these cells were cultured for an additional four days and then exposed to CRH. Like adult rat Leydig cells from work performed by this lab [31], CRH exposure alone did not affect steroidogenesis, but CRH did inhibit hCG-induced steroidogenesis [43]. The reason for the discrepancy between our *ex vivo* fetal testis data and those from Ulisse et al. [43] are unknown but could be related to differences in fetal age at analysis, the different durations of the culture periods, and/or using dispersed fetal Leydig cells versus intact fetal testes. The data described here are the first to examine intact fetal testes from rat and mouse, and the first to show CRHR1 agonism stimulates fetal testis testosterone production.

Similar to our rodent fetal testis data, CRH exposure of MA-10 cells stimulated steroidogenic gene expression and progesterone production (MA-10 cells do not produce testosterone) [34]. The MA-10 cell line was treated with 10 nM CRH for multiple time intervals to determine optimal exposure time (1, 3, 6, or 24 hours). qRT-PCR analysis of *Cyp11a1*, *Cyp17a1*, *Scarb1*, and *Star* showed increased expression of these genes at multiple time points, with all genes increased at 6 hours. When treated with varying CRH concentrations for 6 hours in culture, steroidogenic mRNA levels showed significant increases at 10 nM and 100 nM CRH. Combined with the fetal mouse testis ISH data showing *Crhr1* expression in fetal Leydig cells, the ability of CRH to increase MA-10 steroidogenesis indicates that CRH stimulates fetal testis steroidogenesis by direct activation of Leydig cells.

Multiple organs were screened using qRTPCR to identify potential organ sources for CRH and UCN1 in the rodent fetus (Table 3). *Ucn1* mRNA was undetectable in all organs screened, but *Crh* mRNA levels were detectable in brain and hypothalamus. This is consistent with ISH data from other labs showing *Crh* mRNA is present in the hypothalamus of fetal rats beginning around GD17 to GD21 [51], [52], and *Crh* expression is seen in the human fetal hypothalamus as early as GW12 [53]. At all gestation ages examined, no *Crh* or *Ucn1* mRNA was observed in the fetal testis. The presence of CRH in amniotic fluid from GD17 male rats and GD15 male mice (Figure 8) suggests the rodent fetus produces CRH peptide during the masculinization programming window. Beginning around GW8–10, expression of CRH by the human placenta also coincides with the start of steroidogenesis in the human fetal testis [54], and CRH is detectable in human amniotic fluid during pregnancy [55], [56]. From these data, we conclude that CRH is expressed in the mammalian fetus during the masculinization programming window and that the likely source of CRH in the rodent is the hypothalamus.

Our data show CRHR1 agonism stimulates rodent fetal Leydig cell steroidogenesis under *ex vivo* conditions, but the *in vivo* significance of these data is unknown. The microarray and ISH data indicate *Crhr1* mRNA is expressed in fetal mouse Leydig cells from the early stages of steroidogenesis (GD13 and later). CRHR1 agonists stimulated mouse and rat fetal testis steroidogenesis during the *in utero* masculinization programming window. However, male *Crhr1* knockout mice are fertile [57], [58], suggesting CRHR1 may not be required for *in utero* masculinization of the mouse male reproductive tract. Nonetheless, fetal Leydig cell function has not been analyzed rigorously in *Crhr1* knockout mice, and it remains possible that fetal Leydig cell steroidogenesis is reduced in these mice but not to a level that would cause masculinization defects resulting in infertility.

Some aspects of human male reproductive tract masculinization are independent of LHCGR-mediated stimulation of fetal Leydig cell testosterone production. In males with an inactivating LHCGR mutation, two androgen-dependent tissues (the epididymis and vas deferens) still masculinize [59]. These data suggest that either basal levels of testosterone production are sufficient to masculinize Wolffian duct-derived tissues or that factors other than LHCGR can stimulate fetal Leydig cell steroidogenesis during the initial period of fetal male masculinization (from approximately GW6 to GW10). One possible mechanism is functional removal of a steroidogenic repressor molecule, as has been suggested to occur in rodent fetal testes [60]. Although no human data exist to support such a role, another possible mechanism is steroidogenic stimulation via CRHR1 agonism.

## Supporting Information

Figure S1 mRNA expression levels of select known Leydig- (*Star* and *Cyp17a1*), Sertoli- (*Ptgds* and *Defb19*), and gonocyte- (*Mael* and *Dppa4*) specific genes in mouse GD13 *Mafb*+, *Pou5f1*+, and *Sox9*+ cell isolates. The *Pou5f1*+ cell isolate contained high levels of gonocyte-specific gene mRNA but only background mRNA levels of Leydig- or Sertoli-specific genes. Likewise, only background mRNA levels of gonocyte genes were found in *Mafb*+ or *Sox9*+ cell isolates. Both the *Mafb*+ and *Sox9*+ cell isolates were enriched for Leydig- and Sertoli-specific genes, respectively. However, mRNA levels of Leydig cell-specific genes were above background in the *Sox9*+ cell isolate, and the same was true for Sertoli-specific genes in the *Mafb*+ cell isolate. Thus, while the *Mafb*+ and *Sox9*+ cell isolates were highly enriched in the expected cell population there was some contamination of other somatic cell types but not gonocytes in these two cell isolates. Values shown are the means ± SD. *FDR-corrected p-value <0.05 compared to expression in the expected cell type.
(TIF)

Figure S2 Microarray expression data of known Leydig cell-specific genes (*Cyp17a1*, *Star*, and *Lhcgr*) and selected Leydig cell candidate genes in various mouse testis isolates. A) Expression in mouse *Mafb*+ cells at GD11, GD12, and GD13. B) Expression in mouse GD13 *Mafb*+, *Pou5f1*+, and *Sox9*+ cell isolates. C) Expression in whole mouse testis GD11, GD12, and GD13. GD13 mRNA levels in A and B and GD14 mRNA levels in C were set to 1, and all other data were expressed relative to this value. Values shown are the means ± SD. *FDR-corrected p-value <0.05; ^#^FDR-corrected p-value <0.25.
(TIF)

Figure S3 Microarray expression data of known Leydig cell-specific genes and selected Leydig cell candidate genes in dibutyl phthalate-exposed GD19 rat testis and GD13 mouse ovary and testis *Mafb*+ cell isolates. A) mRNA levels in GD19 rat testis after exposure to dibutyl phthalate. Data for vehicle controls (C) were set to 1 and all values expressed relative to vehicle control. Acute exposures were 1 hr (1), 3 hr (3), 6 hr (6), and 18 hr (18). The subchronic (SC) exposure was a daily exposure from GD12 to GD19. Data for *Vgll3* and *Itih5* are not shown because these genes are not present on the Affymetrix Rat 230 2.0 micorarray chip. B) mRNA levels in GD13 mouse ovary and testis *Mafb*+ cell isolates. Testis values were set to 1 and ovary values expressed relative to the testis values. Values shown are the means ± SD. *FDR-corrected p-value <0.05; ^#^FDR-corrected p-value <0.25.
(TIF)

File S1 Microarray data associated with genes at each step of the comparative genomics process shown in Figure 1. The values shown in the various worksheets are normalized average expression values (AvgSig), fold change (FC) values, and false discovery rate (FDR)-corrected p-values.
(XLSX)

File S2 All genomics data from fetal mouse testis *Mafb*+, *Sox9*+, and *Pou5f1*+ cell isolates. The microarray platform was Affymetrix Mouse Gene 1.0 ST. Datasets used for normalization and statistical analysis are separated by a red line. The values shown are normalized average expression values (AvgSig), fold change (FC) values, and false discovery rate (FDR)-corrected p-values.
(XLSX)

File S3 All genomics data from whole GD11, GD12, and GD14 mouse testes. The microarray platform was Affymetrix Mouse Genome 430 2.0. The values shown are normalized average expression values (AvgSig), fold change (FC) values, and false discovery rate (FDR)-corrected p-values.
(XLSX)

File S4 All genomics data from whole fetal mouse and rat testes after dibutyl phthalate exposure. The microarray platforms were Affymetrix Mouse Genome 430 2.0 and Affymetrix Rat Genome 230. Mouse and rat data are shown in separate worksheets. The values shown are normalized average expression values (AvgSig), fold change (FC) values, and false discovery rate (FDR)-corrected p-values.
(XLSX)

Table S1 Template and primer sequences used in PCR to generate ISH clones for probe generation.
(DOCX)

Table S2 Taqman assays used in qRT-PCR.
(DOCX)
