# Supplementary figures and images for: A Transcriptome-Wide Screen for mRNAs Enriched in Fetal Leydig Cells: CRHR1 Agonism Stimulates Rat and Mouse Fetal Testis Steroidogenesis
Source: PLoS One. 2012 Oct 25;7(10):e47359. doi: 10.1371/journal.pone.0047359 (PMC3484991; doi:10.1371/journal.pone.0047359)

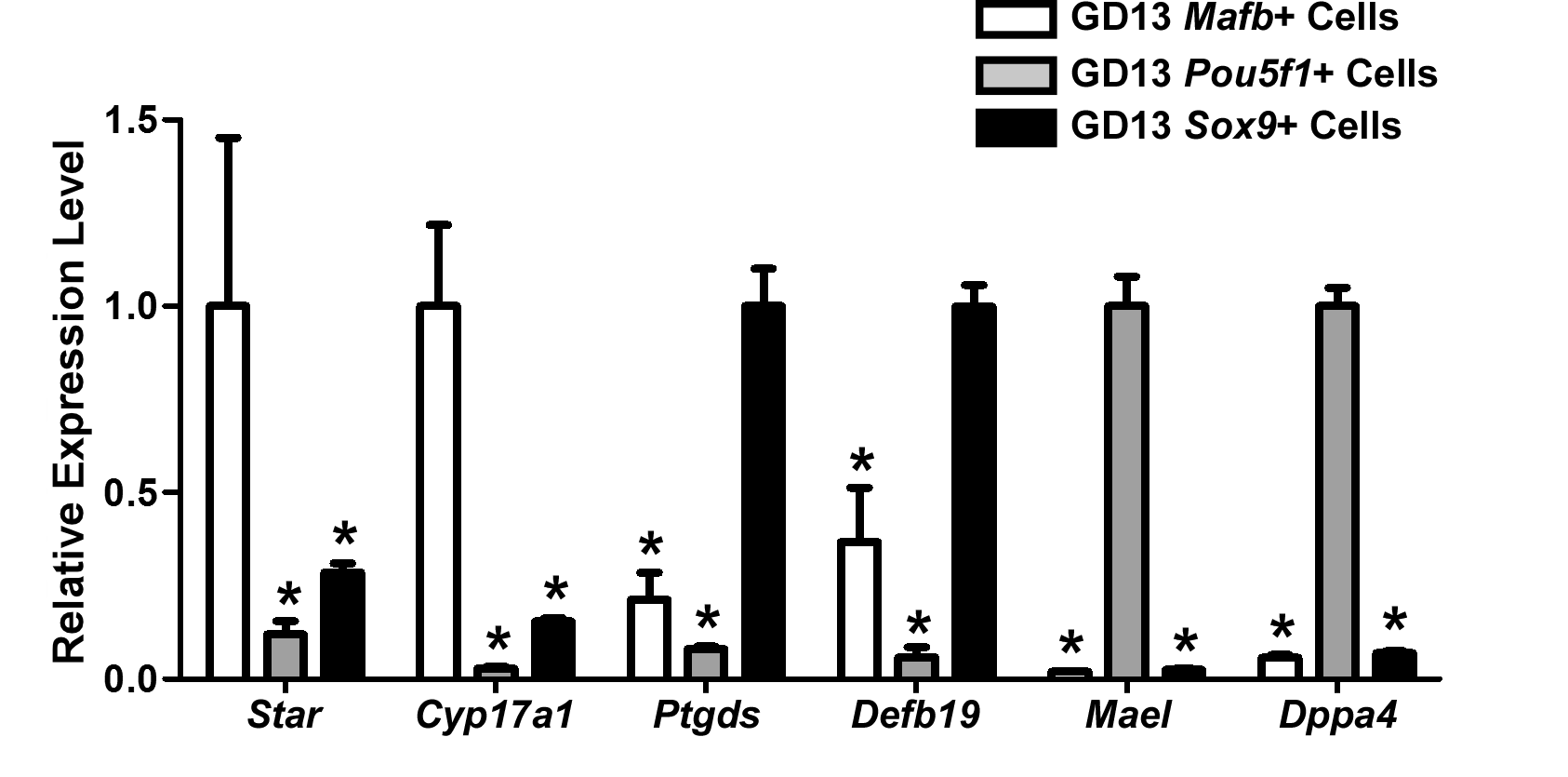

Supplement: Figure S1 — mRNA expression levels of select known Leydig- (Star and Cyp17a1), Sertoli- (Ptgds and Defb19), and gonocyte- (Mael and Dppa4) specific genes in mouse GD13 Mafb+, Pou5f1+, and Sox9+ cell isolates. The Pou5f1+ cell isolate contained high levels of gonocyte-specific gene mRNA but only background mRNA levels of Leydig- or Sertoli-specific genes. Likewise, only background mRNA levels of gonocyte genes were found in Mafb+ or Sox9+ cell isolates. Both the Mafb+ and Sox9+ cell isolates were enriched for Leydig- and Sertoli-specific genes, respectively. However, mRNA levels of Leydig cell-specific genes were above background in the Sox9+ cell isolate, and the same was true for Sertoli-specific genes in the Mafb+ cell isolate. Thus, while the Mafb+ and Sox9+ cell isolates were highly enriched in the expected cell population there was some contamination of other somatic cell types but not gonocytes in these two cell isolates. Values shown are the means ± SD. *FDR-corrected p-value <0.05 compared to expression in the expected cell type. (TIF) [file pone.0047359.s001.tif]

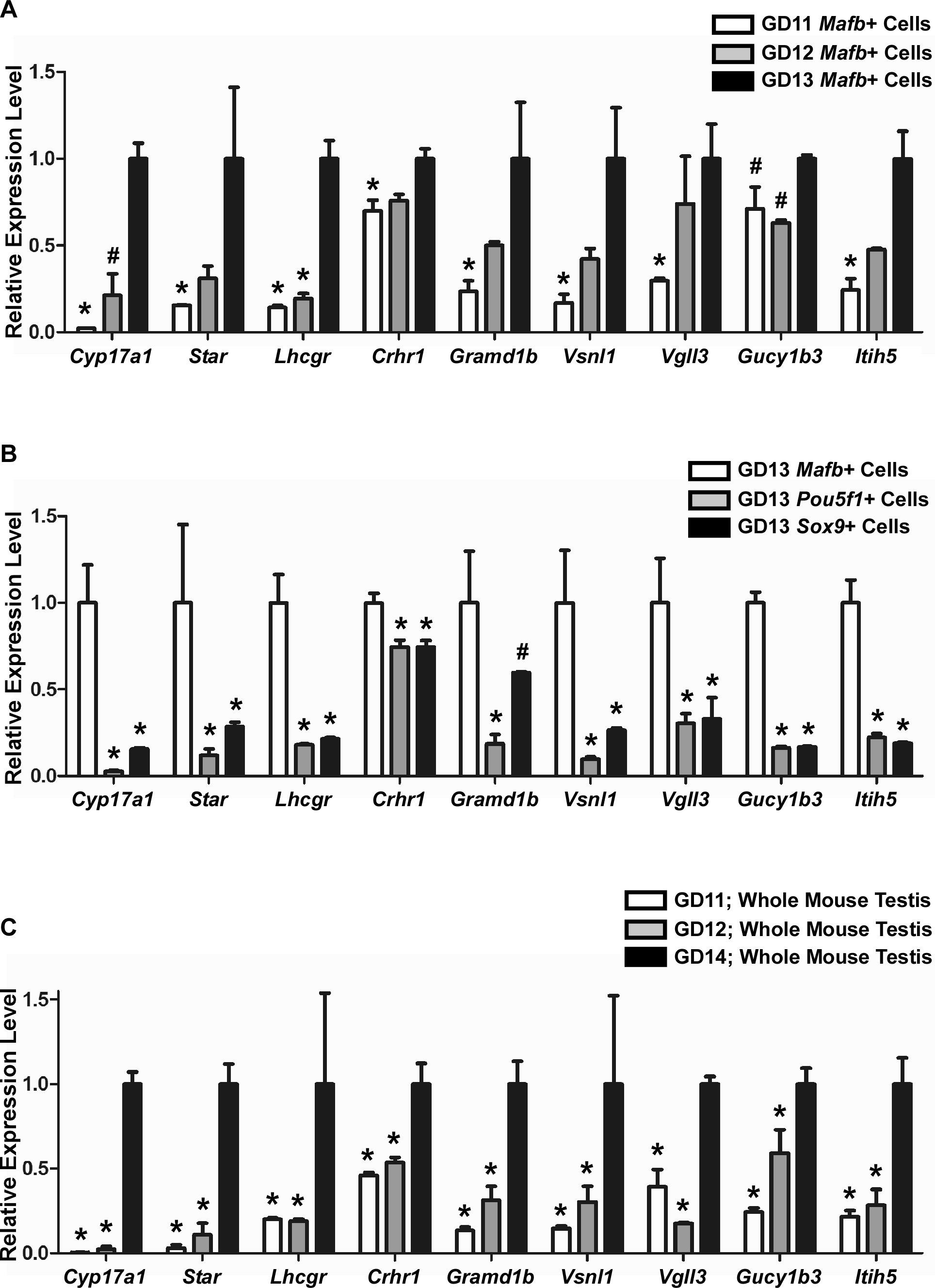

Supplement: Figure S2 — Microarray expression data of known Leydig cell-specific genes (Cyp17a1, Star, and Lhcgr) and selected Leydig cell candidate genes in various mouse testis isolates. A) Expression in mouse Mafb+ cells at GD11, GD12, and GD13. B) Expression in mouse GD13 Mafb+, Pou5f1+, and Sox9+ cell isolates. C) Expression in whole mouse testis GD11, GD12, and GD13. GD13 mRNA levels in A and B and GD14 mRNA levels in C were set to 1, and all other data were expressed relative to this value. Values shown are the means ± SD. *FDR-corrected p-value <0.05; #FDR-corrected p-value <0.25. (TIF) [file pone.0047359.s002.tif]

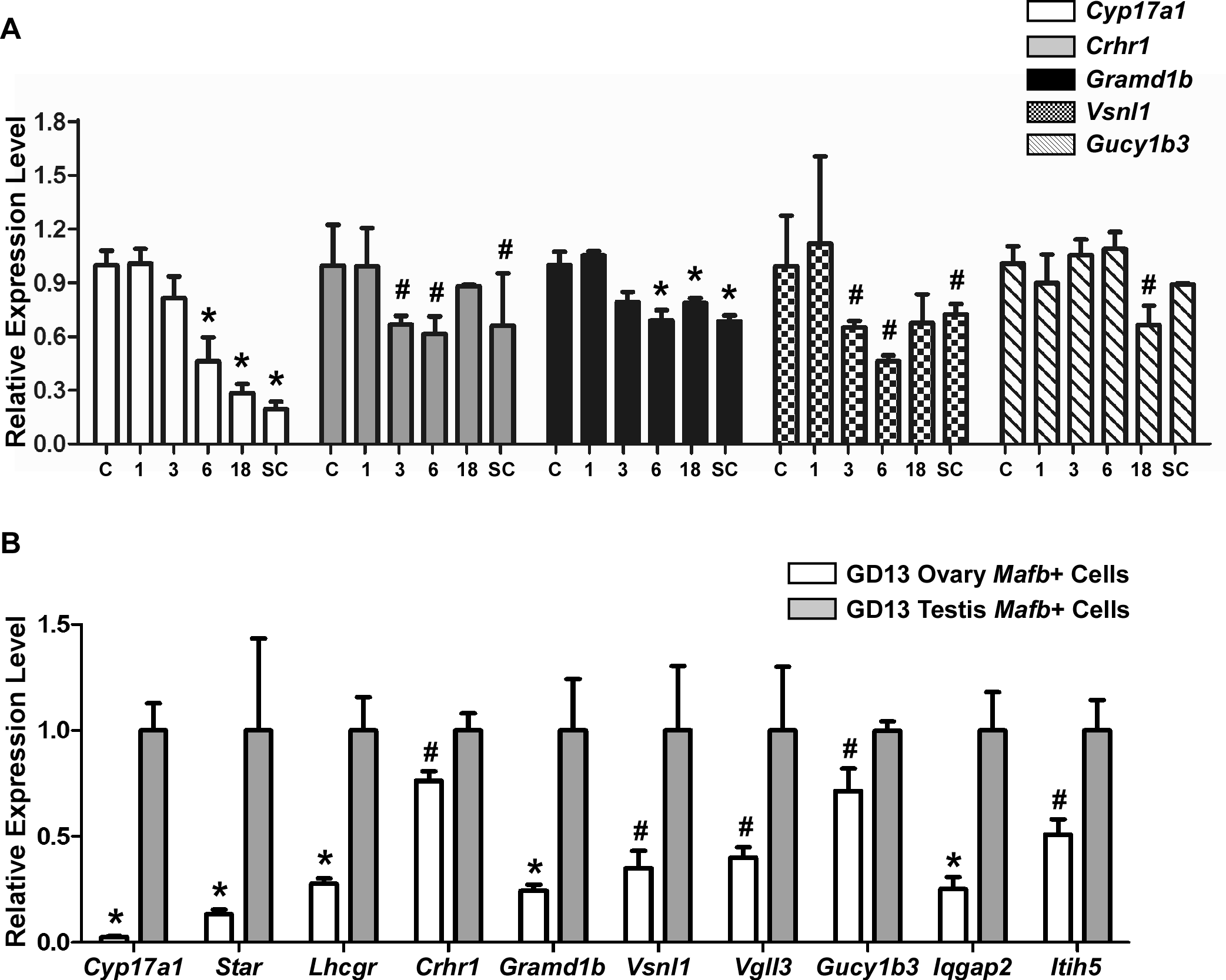

Supplement: Figure S3 — Microarray expression data of known Leydig cell-specific genes and selected Leydig cell candidate genes in dibutyl phthalate-exposed GD19 rat testis and GD13 mouse ovary and testis Mafb+ cell isolates. A) mRNA levels in GD19 rat testis after exposure to dibutyl phthalate. Data for vehicle controls (C) were set to 1 and all values expressed relative to vehicle control. Acute exposures were 1 hr (1), 3 hr (3), 6 hr (6), and 18 hr (18). The subchronic (SC) exposure was a daily exposure from GD12 to GD19. Data for Vgll3 and Itih5 are not shown because these genes are not present on the Affymetrix Rat 230 2.0 micorarray chip. B) mRNA levels in GD13 mouse ovary and testis Mafb+ cell isolates. Testis values were set to 1 and ovary values expressed relative to the testis values. Values shown are the means ± SD. *FDR-corrected p-value <0.05; #FDR-corrected p-value <0.25. (TIF) [file pone.0047359.s003.tif]
