# Supplementary material for: A Transcriptome-Wide Screen for mRNAs Enriched in Fetal Leydig Cells: CRHR1 Agonism Stimulates Rat and Mouse Fetal Testis Steroidogenesis
Source: PLoS One. 2012 Oct 25;7(10):e47359. doi: 10.1371/journal.pone.0047359 (PMC3484991; doi:10.1371/journal.pone.0047359)
Supplement: Table S1 — Template and primer sequences used in PCR to generate ISH clones for probe generation. (DOCX) [file pone.0047359.s008.docx]

| **Gene** | **Template** | **Forward Primer (5’🡪3’)** | **Reverse Primer (5’🡪3’)** |
| --- | --- | --- | --- |
| *Crhr1* | Accession # BC103675 | GTGAGAGCCTGTCCCTGG | GGGGCAAGAGCACCAG |
| *Gramd1b* | Accession # BC070451 | TAGATGCTGAAGTCCTCACCCAC | AGCGAGTCCTTCATCTGGTCC |
| *Itih5* | GD15 mouse testis | GGGTTGCCCCTCTTCTCAG | TGTCACCAATGTTCTGCTCTCTC |
| *Vgll3* | GD15 mouse bladder | GAGTACCTTAACTCTCGCTGTGT | GTCCTGATGCTGAAGACCTGT |
| *Vsnl1* | Accession # BC046226 | ACCTGGTGAAGAGCACTGAG | CAATGGAAGGGTCGCTT |

**Templates and primers for generating *In situ* hybridization clones^1^**

^1^ *Cyp11a1* ISH used a previously described probe Yao HH, W Whoriskey, et al. (2002). “Desert Hedgehog/ Patched 1 signaling specifies fetal Leydig cell fate in testis organogenesis.” Genes Dev **16**(11): 1433-40.
