# Supplementary material for: A Transcriptome-Wide Screen for mRNAs Enriched in Fetal Leydig Cells: CRHR1 Agonism Stimulates Rat and Mouse Fetal Testis Steroidogenesis
Source: PLoS One. 2012 Oct 25;7(10):e47359. doi: 10.1371/journal.pone.0047359 (PMC3484991; doi:10.1371/journal.pone.0047359)
Supplement: Table S2 — Taqman assays used in qRT-PCR. (DOCX) [file pone.0047359.s009.docx]

| **Gene Symbol** | **Mouse Assay** | **Rat Assay** |
| --- | --- | --- |
| *Crh* |  | Rn01462137_m1 |
| *Cyp11a1* | Mm00490735_m1 | Rn00568733_m1 |
| *Cyp17a1* | Mm00484040_m1 | Rn00562601_m1 |
| *Scarb1* | Mm00450234_m1 | Rn00580588_m1 |
| *Star* | Mm00441558_m1 | Rn00580695_m1 |
| *Tbp* | Mm00446973_m1 | Rn01455646_m1 |
| *Ucn1* |  | Rn00569682_m1 |

**Taqman assay for qRT-PCR**
